# Exploring user experience and performance of a tedious task through human–agent relationship

**DOI:** 10.1038/s41598-023-29874-5

**Published:** 2023-02-21

**Authors:** Chao Zhou, Yulong Bian, Shu Zhang, Ziyang Zhang, Yaoyuan Wang, Yong-Jin Liu

**Affiliations:** 1 Department of Computer Science and Technology, Tsinghua University, Beijing, 100084 China; 2 BNRist, MOE-Key Laboratory of Pervasive Computing, Tsinghua University, Beijing, 100084 China; 3 Advanced Computing and Storage Laboratory, Huawei Technologies Co Ltd, Shenzhen, 518000 China

**Keywords:** Human behaviour, Psychology, Engineering

## Abstract

Positive human–agent relationships can effectively improve human experience and performance in human–machine systems or environments. The characteristics of agents that enhance this relationship have garnered attention in human–agent or human–robot interactions. In this study, based on the rule of the persona effect, we study the effect of an agent’s social cues on human–agent relationships and human performance. We constructed a tedious task in an immersive virtual environment, designing virtual partners with varying levels of human likeness and responsiveness. Human likeness encompassed appearance, sound, and behavior, while responsiveness referred to the way agents responded to humans. Based on the constructed environment, we present two studies to explore the effects of an agent’s human likeness and responsiveness to agents on participants’ performance and perception of human–agent relationships during the task. The results indicate that when participants work with an agent, its responsiveness attracts attention and induces positive feelings. Agents with responsiveness and appropriate social response strategies have a significant positive effect on human–agent relationships. These results shed some light on how to design virtual agents to improve user experience and performance in human–agent interactions.

## Introduction

An intelligent agent is a software entity that can conduct operations continuously and autonomously after sensing a specific environment^1^. As AI technology rapidly progresses and becomes more widespread, intelligent agents have started appearing more frequently in our daily lives. The co-existence of people and these agents significantly promotes the study of human–agent interaction^2^. Agents are often given roles (for example, the roles of the teacher and partner^3^) based on which agents interact with people and assist people through virtual activities^4^.

User experience and performance are important factors in evaluating human–agent interaction^2,5,6^. Thus far, most existing human–agent interaction studies have focused on task performance efficiency without considering the human–agent relationship^7,8^. Despite achieving high-performance efficiency, forcing people to adhere to the repetitive operations of the machine can cause fatigue and a poor user experience^9–11^. In this study, we investigate user experience and performance in human–agent interaction using a tedious task from the perspective of positive human–agent relationships.

To the best of the authors’ knowledge, “tedious tasks” have not yet been clearly defined in the literature. Based on the description of tedious activity in previous literature^9,10^, we define a “tedious task” as an activity or piece of work that the processes are unnecessarily monotonous, repetitive, and time-consuming. Performing a tedious task in human–agent interaction easily leads to a negative user experience^9,10^, and even reduces human performance and motivation to participate in the task. Therefore, it is desirable to explore effective factors to improve the human experience in tedious tasks by establishing a positive human–agent relationship.

Pedagogical agents have been widely studied in the literature as special agents. The presence of an animated pedagogical agent can lead to positive effects in learning activities, which has been well known as *Persona Effect*^12,13^. Furthermore, existing research shows that not only the presence of a pedagogical agent (whether there is an agent or not) but also the cues of an agent (the characteristics of an agent), such as appearance, politeness, feedback, behavior, emotions, and personality, can evoke a positive effect^8,14–17^. Inspired by the persona effect rule, we expect positive results to extend to tedious tasks. In this study, we investigate this issue using virtual agents and a tedious task to induce positive outcomes in human–agent interactions. In particular, we focus on human likeness and responsiveness, which are important attributes of agents studied in previous works; that is, they can activate human social cognition, feelings, and behavioral responses to agents during interaction^18–21^. Especially for responsiveness, we think it is important to give agents the ability to show social responsiveness, and make them adopt proper strategies to respond to humans, which may beneficial to human–agent interaction. However, their effects on human–agent relationships and performance remain unclear, especially in tedious activities. Therefore, we focused on human likeness and responsiveness as key social cues of agents and studied their effects on the human–agent relationship and performance.

**Figure 1:**
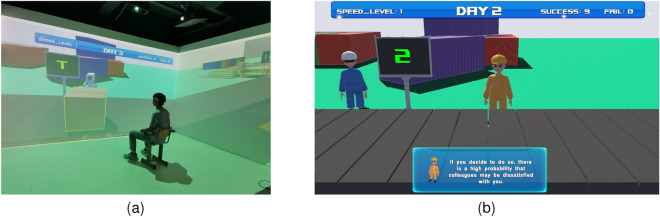
Our prototype system is in which participants experience a tedious task with a virtual partner (i.e., an agent) in an immersive virtual environment. (**a**) A user is playing with the prototype system. (**b**) A virtual agent is designed as a partner. See accompanying demo Video for more details.

In this study, we designed a tedious task by simulating a parcel-sorting assembly line in an immersive virtual environment (IVE). We used this task to build an experimental environment in which user experience and performance in a tedious task were explored (Fig. 1). In the designed experimental environment, an agent was used as the participant’s virtual partner. In particular, by controlling the agent’s appearance, verbal features, and intelligent feedback, the effects of two social cues of the agent were explored: human-likeness (how much an agent resembles a human) and responsiveness (how an agent responds to humans). Regarding responsiveness, we not only examined the effect of its presence (Experiment 1) but also considered the effect of different social response strategies (Experiment 2). This study systematically examined the potential effects of these cues on the human–agent relationship and user performance in a tedious task. The contributions of this study are as follows.

- An elaborate VR environment is designed to study user experience in tedious tasks through human–agent relationships.
- The effects of an agent’s human likeness and responsiveness on human–agent relationships and user performance in a tedious task are examined in the VR environment.
- The effects of an agent’s human-likeness and social response strategy on human–agent relationships and user performance in a tedious task were examined.

The remainder of the paper is organized as follows. We briefly review related work in “Related works” section. Then we present our developed tedious task in an IVE in “Tedious task construction by sorting parcels on the virtual assembly line” section and present two empirical experiments in “Experiment 1: effect of agent’s human-likeness and responsiveness in tedious tasks” and “Experiment 2: exploring the effect of social response strategy in agent’s responsiveness” sections, respectively. Finally, we discuss main findings of our studies in “General discussion” and conclude our work in “Conclusions”.

## Related works

### Human–agent relationship

Intelligent agents are becoming increasingly widespread, which promotes the study of human–agent interaction^2^. Studies have proposed that effective human–agent interaction promotes positive motivational, behavioral, and cognitive outcomes for increased task performance efficiency^8,22,23^. The positive outcomes of human–agent relationships have also been concerned.

The human–agent relationship refers to the social and emotional aspect of human–agent interactions. Few studies have investigated the relationship between human users and several types of computer agents. In early studies of human–agent relationships, Bickmore et al. focused on agents designed to establish and maintain long-term social-emotional relationships with human users^24^. They found that compared to agents without any deliberately designed social-emotional or relationship-building abilities, agents with these abilities obtain more respect, likeness, and trust from human participants. Recent studies have explored methods to improve an agent’s ability to establish relationships with users and further confirm the effectiveness of this ability^5,25^. Therefore, improving human–agent relationships can improve human experience and performance in human–agent interactions, leading to positive changes in behavioral, cognitive, or emotional states.

### Study in tedious activities

People often have to engage in tedious activities in various scenarios, and many of these activities are related to interactions, such as selecting targets in visual navigation^26^. Tedious tasks easily lead to fatigue and poor user experience^9–11^ and even reduce people’s motivation (to participate in activities) and performance. Interactive learning activities can prove ineffective if the tasks are tedious and time-consuming, because fatigue and frustration may negate the positive learning effect, leading to poor motivation in subsequent sessions^27^.

One way to address tedious tasks is to automate and intelligently design technologies to replace manual labor. For example, manually detecting depression is a time-consuming and tedious task. Here, Ay et al. developed a fully automated depression diagnosis system by analyzing electroencephalogram (EEG) signals^28^. Other studies addressed tedious work by optimizing the designs^27,29^. An example presented in^27^ was that Tom Sawyer introduced a design concept that transforms image labeling and other tedious tasks into entertainment through redesigning tasks. However, in many practical situations, automated methods are difficult or even disallowed, and the cost of redesigning tasks is high. It is essential to find effective methods to enhance the user experience without altering the primary tasks. In this study, we explore these methods by establishing a positive human–agent relationship in a tedious task.

### Persona effect and social cues of agent

Using agents in a virtual environment is a typical element and an added value that has a significant impact on user experience^4^. The positive effect of virtual agents occurring in an interactive virtual learning environment has been studied and is known as the persona effect. Persona Effect is defined as that *the presence of a life-like character in an interactive learning environment—even if they are not expressive—can have a strong positive impact on student’s perception of their learning experience*^13^.

Designing appropriate cues (especially social cues) for agents helps to induce the persona effect. Previous studies demonstrated that agents’ social cues (for example, human likeness, role, dress, sociality, emotion, and personality) have positive effects on learners’ mental and behavioral outcomes, such as social judgements, interest to learn, self-efficacy, and learning performance^7,8,15,16,30,31^.

To interpret the persona effect, the *Media Equation* theory proposed by Reeves et al. was commonly used, which states that people exhibit remarkable social reactions to computers and other media (including agents) and treat them as real people, even if they have actual real feelings, intentions or human motivations^32^. When a computer or robot presents a set of cues (such as language and behaviors) that would normally be associated with humans, humans respond by exhibiting social behaviors and making social attributions^33^. Therefore, human–agent interaction reflects social cues prevalent in human–human interaction, and positive effects in human–human interaction can occur to a certain extent^22,30^.

Inspired by the persona effect, we expect the positive effects of an agent to sustainably exist in human–agent relationships. For these agents to function properly, they require social cues to exhibit a certain amount of social skills or sociality^5,34^. As mentioned above, human likeness and responsiveness are two important attributes of agents that have been studied previously. Human-likeness is a social cue for a human’s social response to an agent or a robot^18^. A key characteristic of human likeness is appearance. The appearance reflects the shape of the body and the degree to which it resembles the human body^35,36^. Responsiveness is another social cue that facilitates interpersonal interaction^20^. These cues can activate human social cognition, feelings, and behavioral responses to agents during interaction^18–21^.

Based on previous studies, human likeness and responsiveness are expected to play a positive role in human–agent relationships and performance in human–agent interaction tasks. However, their impact on these aspects remains unclear, especially in tedious activities. Therefore, this study considers human likeness and responsiveness as two key social cues of agents to study their effects on human–agent relationships and performance by simulating parcel sorting on an assembly line in an immersive virtual environment (IVE) as a tedious task.

## Tedious task construction by sorting parcels on the virtual assembly line

Several tedious tasks have been described in previous studies. However, to the best of our knowledge, there is still a lack of a typical tedious task paradigm or platform for user experience studies. In this study, we constructed a tedious task in an IVE. Details are as follows.

### Task and design scenario

To identify an appropriate scenario for a tedious task, we conducted brief interviews with five college students followed by a literature review. In the interview, interviewees mentioned that they had once worked as interns in a nearby factory during summer vacation, and they all reported that sorting items repeatedly for a long time on an assembly line is a tedious task. One interviewee stated that *some of my classmates and I once went to a factory to do an internship on the assembly line. One week later, we all quit. The work was simple but extremely time-consuming, repetitive, and tedious.* Our literature review also showed that tasks on an assembly line are generally tedious, repetitive, and time-consuming^37^.

Furthermore, we found that using a tedious task on the assembly line is suitable for setting up agents with controllable cues; thus, we used this scenario for our study.

### Construction of the prototype system

#### Software of the system

**Figure 2:**
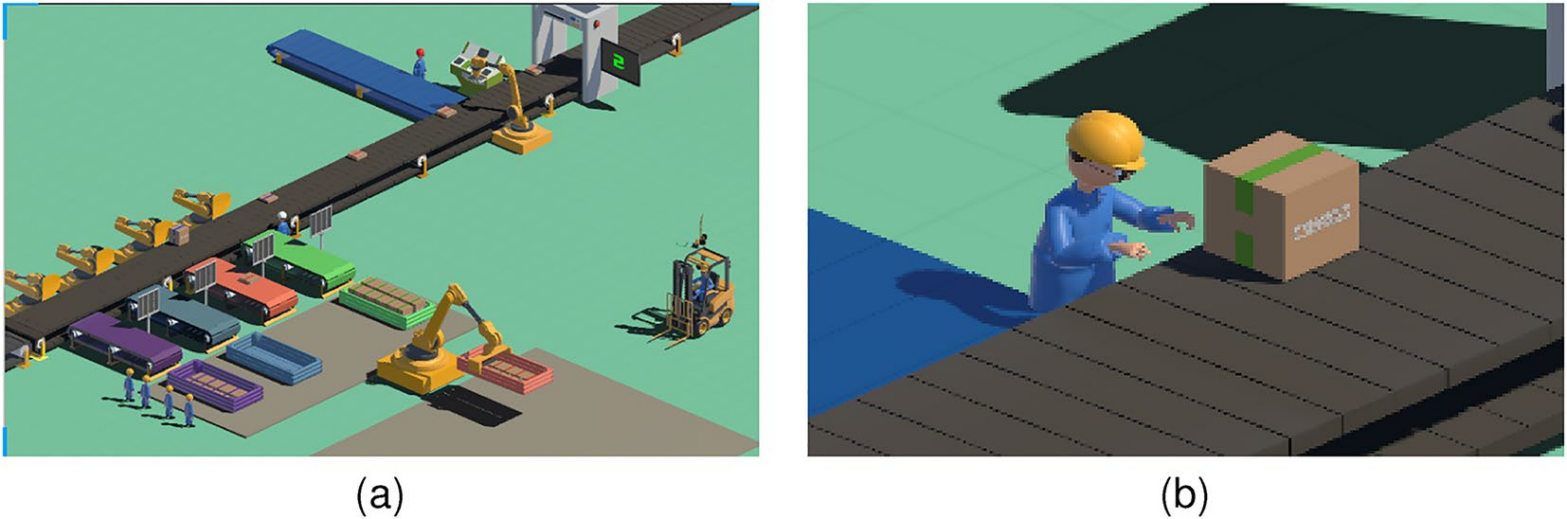
Our prototype system is in which participants experience a tedious task with a virtual partner (i.e., an agent) in an IVE. (**a**) A user is playing with the prototype system. (**b**) A virtual agent is designed as a partner. See accompanying demo Video for more details.

A prototype system of a virtual assembly line for parcel sorting was developed using the Unity 3D 2019 software. Two snapshots of the virtual assembly line are shown in Fig. 2.

*Task* The user was designed to be an intern who participated in the internship. During the internship, the user was required to manually complete the task of sorting parcels (Fig. 2b). Many parcels (including five types) were transported at a certain frequency on the assembly line. The parcel can be successfully sorted only by pressing the corresponding number keys in a timely and accurate manner. The user’s performance was displayed on the screen in the IVE in real time.

*Events* In addition to sorting parcels, the user must deal with several events during the internship experience. These events were designed to simulate real-world situations encountered during daily activities. Users were instructed to select an action from the preset options when addressing these events.

*Agent* The agent’s role was designed as that of the user’s partner. The agent was not designed to help users complete the task directly but to provide suggestions and feedback based on the user’s behavior. The appearance, voice, and feedback of the agent can be manipulated flexibly.

#### Hardware of the system

**Figure 3:**
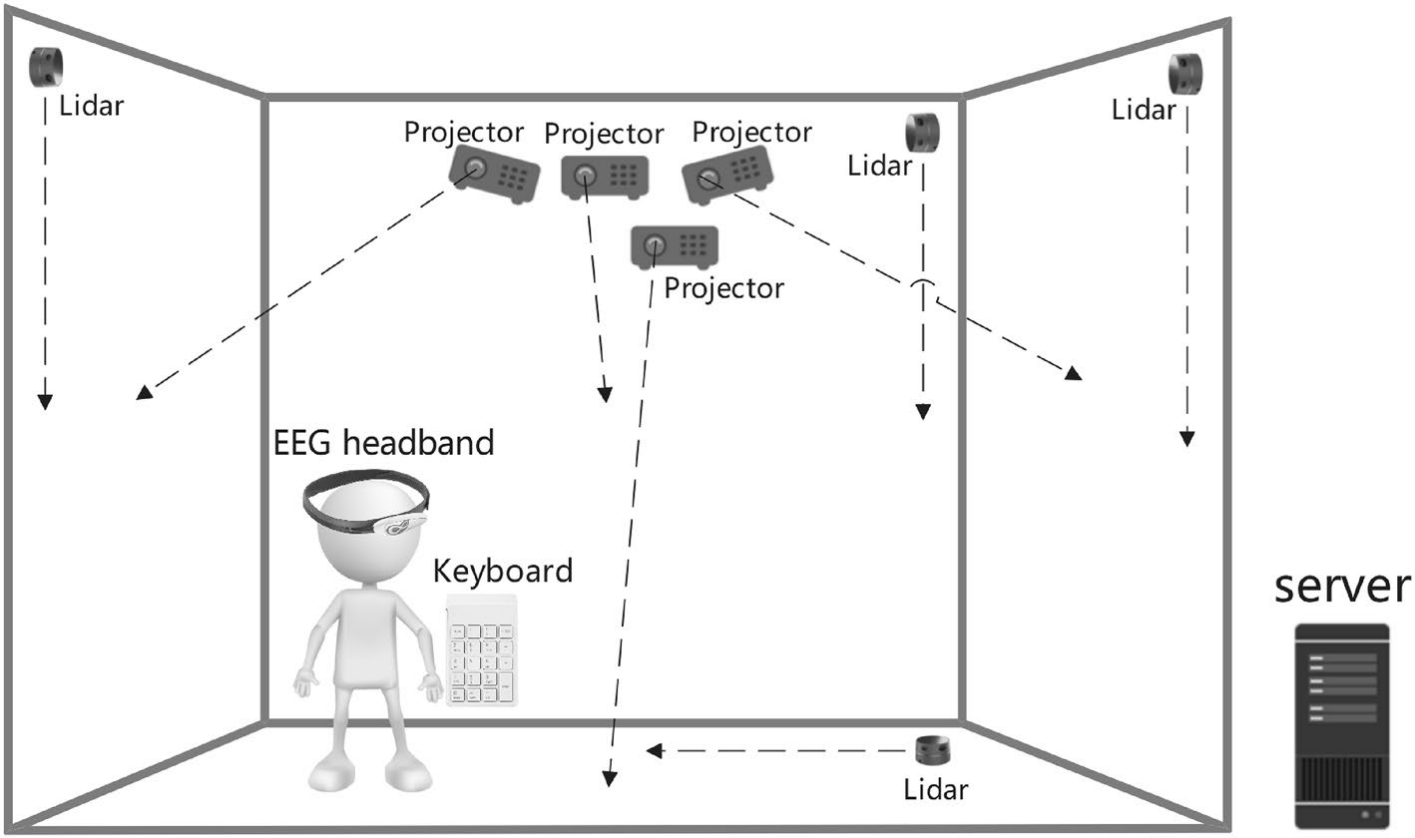
Overall framework of the system hardware.

To ensure a highly immersive experience, the system was built into an interactive IVE. The overall framework of the system hardware is shown in Fig. 3. The details of the components are described below.

*Display devices* The system was displayed in a cave automatic virtual environment (CAVE)^38^, where virtual contents were projected onto four surfaces (three walls and the floor) of a room-sized cube to represent a specific pre-designed IVE.

*Interactive devices* Each of the four surfaces projected in the CAVE was equipped with a 360$^\circ$ laser range finder, YDLIDAR G2. These laser range finders scan at 5–12 Hz and range from 0.1–12 m, enabling multitouch interaction on all four surfaces. During the task, the user selects actions by directly touching virtual content on the surface. The touch position was identified by using laser rangefinders.

**Figure 4:**
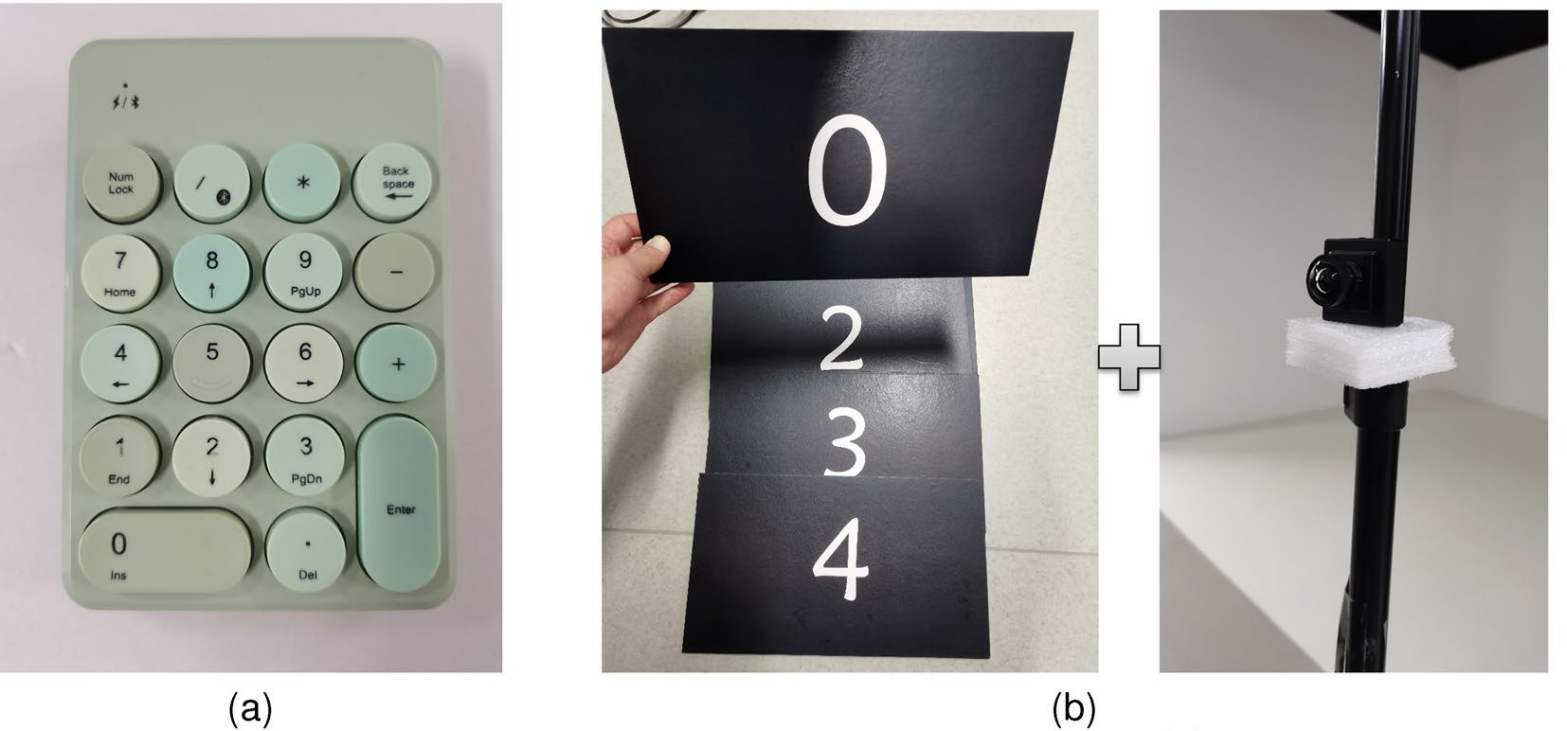
Some key components are used in the system hardware. (**a**) Bluetooth keyboard. (**b**) Physical boards (with five numbers) and an RGB camera.

The system provides two interactive ways to complete the parcel-sorting task. One way is to use a Bluetooth keyboard to ensure that users can press numbers directly (Fig. 4a). The other method is using an RGB camera with the aid of machine vision algorithms to identify the number on the physical cardboard that the user has picked up (Fig. 4b). The latter method can provide a higher level of physical challenges than the former^39^.

*Attention evaluation device* We used a portable EEG headband (Brainlink Pro) equipped with a NeuroSky ThinkGear sensor^40^ to assess the sustained attention level of users. It has received authorization from the U.S. Federal Communications Commission and has been approved for use in the European Union^41^. The NeuroSky ThinkGear is a convenient non-invasive biosensor that has been widely used in brain-computer interface systems^42,43^. It connects to three dry EEG electrodes and comprises three dry EEG electrodes (i.e., active, reference, and ground), placed on the forehead using an elastic band. The dry EEG electrodes were placed close to the standard EEG locations F7, Fp1 (bipolar channel), and Fpz (ground electrode) according to the International 10–20 system. The NeuroSky sensor samples EEG activity at frequencies up to 512 Hz. We used the headband to monitor the user’s EEG signal in real-time and transmit the EEG data to the server via a wireless Bluetooth connection^40^.

The overall environment of the prototype system is shown in Fig. 1a. Based on the constructed prototype system, we conducted two experiments (presented in “Experiment 1: effect of agent’s human-likeness and responsiveness in tedious tasks” and “Experiment 2: exploring the effect of social response strategy in agent’s responsiveness” section) to explore the effects of an agent’s human-likeness and responsiveness on human–agent relationships and performance in the parcel sorting task.

### Usability of the system

To evaluate the performance of our designed system in terms of usability and immersion, we conducted a test using a sample of eight users. Usability was quantified using the system usability scale (SUS), which can identify systems with good/poor usability^44^. Immersion was measured using the immersive experience questionnaire (IEQ), which has shown good reliability and has been widely used in previous VR studies^45,46^. The results indicate that the total SUS score is 89.06 ± 5.28, which means the system is acceptable in terms of acceptability (above 70), and excellent in terms of adjective rating (above 85), suggesting good usability of our system. The immersion scores were then calculated by summing all questions in the IEQ. The IEQ score of our system was 120.75 ± 6.86, which is higher than that of the highly immersive VR system in a previous study (118.3 ± 11.3^46^). These results suggest that the proposed system can provide users with good usability and immersion.

## Experiment 1: effect of agent’s human-likeness and responsiveness in tedious tasks

### Purpose

In this section, we experimented to explore the effect of an agent’s human-likeness and responsiveness on the human–agent relationship and performance in a tedious task, that is, the parcel sorting task summarized in “3Tedious task construction by sorting parcels on the virtual assembly line”.

### Participants

Sixteen undergraduate students (10 males and 6 females) were recruited for this study. Their ages ranged from 19 to 25 years, with an average of 20.31 years (*SD* = 1.49 years). All patients had normal hearing and normal or corrected-to-normal vision. Informed consent was obtained from all the participants. This study was conducted in accordance with the Declaration of Helsinki and approved by the Ethics Committee of Human Experimentation at Jining No.1 People’s Hospital.

### Experimental design

**Figure 5:**
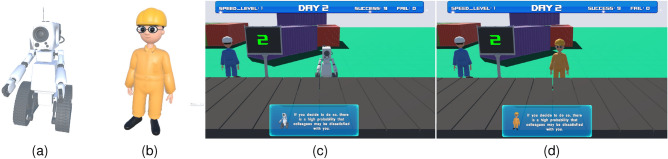
Design of human-likeness and responsiveness of agent in Experiment 1. (**a**) The robot-like virtual agent. (**b**) The human-like virtual agent. (**c**) A snapshot of text suggestions given by a robot-like agent. (**d**) A snapshot of text suggestions given by a human-like agent. See accompanying demo Video for animation details.

Our experiment used a 2 (human-likeness of agent: robot-like/ human-like) $\times$ 2 (responsiveness of agent: with responsiveness/without responsiveness) factorial, within-subject design. Considering that human likeness might significantly affect the human–agent relationship^47,48^, we designed two agents with different human likeness by controlling their appearance and voice:

- Robot-like agent: This virtual agent was designed as the participant’s companion in the form of a robot (Fig. 5a) with a robot-style voice.
- Human-like agent: This virtual agent was designed to be the participant’s companion with a humanoid appearance (Fig. 5b) and a natural human voice.

We designed the responsiveness of agents by controlling their interactive feedback to participants:

- Agent with responsiveness: The agent can actively provide reminders and suggestions in voice or text according to the participant’s operations during the task (Fig. 5c,d).
- Agent without responsiveness: The agent did not provide reminders or suggestions to the participants.

Given the $2\times 2$ factorial within-subject design, there were four types of agents:

- Virtual robot-like agent without responsiveness
- Virtual robot-like agent with responsiveness
- Virtual human-like agent without responsiveness
- Virtual human-like agent with responsiveness

Both factors (human-likeness and responsiveness) of the agents were within-subject factors, and each participant needed to experience all four types. Furthermore, the individual characteristics of the participants remained unchanged in the within-subject design. To avoid the order effect, the order of experience of the four types was counterbalanced using a Latin square design. There were four sequences, and accordingly, the number of participants was a multiple of four. The dependent variables in the design consisted of two aspects: perceived human–agent relationship (perceived closeness, intimacy, and involvement with the agent) and task performance (the accuracy of the task and the attention level), which were analyzed below.

### Apparatus and task

We used the system described in “Construction of the prototype system” section as the experimental environment (Fig. 1a). Note that to identify the parcel type, using physical cardboard selected by participants can simulate the interaction in real sorting to a certain extent, which is physically challenging (i.e., less tedious). To increase the tediousness of the task, we had participants use a Bluetooth keyboard instead of a physical cardboard to complete the task.

The virtual assembly line was projected onto the three vertical screens of the CAVE. In the experimental task of this study, the participants were required to sort 72 parcels (including five types marked with five colors). Parcels were randomly generated every 6 s and transported on the assembly line from left to right. When the package was transported to the middle screen of the CAVE, participants were required to sort the package. The parcel can be successfully sorted only by pressing the corresponding number keys in a timely and accurate manner. After each sorting, the system would timely feedback on “correct” or “wrong” through a notice board in the scene.

The agents had two types of interactions with the user: (1) the agents provided voice feedback according to the dynamic task performance of the user. Specifically, if the user made a mistake in the sorting task, the agent will immediately remind the user “We just made a mistake. Next, we must be more careful. ” If the user continuously and correctly sorts six times, the agent will immediately encourage the user “Well done! We have become increasingly skilled. Keep going.” (2) The agents provide voice and text feedback according to the user’s choice to address the events. Only the agent partner (robot or colleague dressed in yellow) provides the above feedback, while other agents (dressed in blue) do not provide feedback to the user. The specific feedback is presented in Table 1 of the Appendix.

Moreover, the participants were required to handle three events during the task. When each event occurred, the system simulated and showed the virtual scenarios to the participants, and the participants were required to make a behavioral choice from the alternatives. Meanwhile, the process of sorting parcels is paused. For example, one of the events is that “Consider leaving work early.” A colleague offers the participant to drop off unfinished work and leave early to have fun together-this is the scenario of the event. Subsequently, the participant needed to address this event by choosing an action from alternative options, for example, “Give a stern rebuff.” When the participants finished the choice, the agent provided corresponding comments and suggestions on the possible consequences of this decision, for example, “Your work principle is correct, but it may be better if you attempt to use a gentler way first.” The details of these three events are provided in the Appendix.

### Measures

The dependent variables consisted of task performance and the perceived human–agent relationship of the participants. The measures of task performance included the accuracy of the task and attention level during the task. The measures of perceived human–agent relationships include perceived involvement, closeness, and intimacy with agents.

#### Accuracy of task

The system automatically recorded the accuracy of each participant’s task. The recorded data included the number of misclassified and correctly classified parcels in the sorting process, and the sorting accuracy rate was calculated to evaluate the task accuracy.

#### EEG-based attention

Attention refers to the intensity of a user’s level of mental “focus,” such as that which occurs during intense concentration and directed (but stable) mental activity. The proprietary eSense algorithm built into the EEG headband was used to evaluate the player’s attention level based on real-time measured EEG signals. First, the collected EEG signal is pre-processed to remove eye movement artifacts. Then, the eSense algorithm works by performing a Fast Fourier Transform on the collected EEG data, and then the EEG wave are filtered to obtain alpha and beta waves according to the requirements. The power spectrum is analyzed and the basic reference value is selected to complete the normalization. Finally, Attention level is calculated according to the power spectrum of EEG band. That is, the eSense algorithm extracts metrics related to attention, and output attention values (ranging from 0 to 100) at 1 Hz, indicating the intensity of concentration on selective information (whether subjective or objective)^49,50^. The attention values reported by the eSense algorithm have been demonstrated to have strong positive correlations with self-assessment and other physiological measures of attention^42,43,51^. Distractions, mind-wandering, inattention, and anxiety can reduce attention levels. The attention value of each player was recorded during the task.

#### Personal involvement

Personal involvement is operationally defined as important to the person and can be activated by the presence of situational and/or intrinsic self-relevance to an object^52^. This is an important measure of relationships. In our study, involvement refers to participants’ involvement with agents. Thus, involvement is operationalized as the perceived importance and/or self-relevance of virtual agents to the participant. Personal involvement was measured using a 5-item scale adapted from Novak et al.’s work^53^ by Liu et al.^52^. We adapted the scale by adding the context “How do you feel about the agent?” to make the items suitable for measuring personal involvement with agents. All items adopted a seven-point Likert scale, with anchors ranging from 1 (total disagreement) to 7 (total agreement). The reliability of the scale is good ($\alpha =0.845$).

#### Perceived closeness and intimacy in relationship

A sense of closeness and intimacy is essential for building and maintaining relationships, achieved through social interaction^54,55^. Most existing measures of closeness and intimacy were designed to measure daily relationships with humans, such as the emotional closeness questionnaire (ECQ), relational intimacy questionnaire (RIQ)^56^ and Flores et al.’s 5-item questionnaire^57^. They are unsuitable for measuring the current situational experiences with agents. In our study, we used the measure of emotional closeness from China Family Panel Studies (CFPS)^58^. This measure uses a single item on an 11-point scale ranging from 0 (not close at all) to 10 (extremely close) to measure closeness. A single-question measure is commonly used to measure closeness^59^, which can avoid confusion when participants understand an item. Similarly, we used a single item on an 11-point scale to measure perceived intimacy.

### Procedure

At the beginning of the experiment, the participants were given a detailed introduction to the task and how to operate the system. Subsequently, each participant experienced all four types of agents completing the task in a certain order. Each round of the task took approximately 12 min. The attention-level output of the EEG device was recorded. When each type of task was completed, the participants filled out a questionnaire containing the scales in the “Perceived closeness and intimacy in relationship” and “Personal involvement” sections. After the experiment, each participant answered a free-response question to share their experience.

### Results

First, Kolmogorov–Smirnov tests (K–S tests) were performed. Although the sample size was small, the results support the normality assumption of normality ($ps>0.05$). We then performed numerous point-biserial correlations, and the results did not show significant correlations between sex and dependent variables ($ps>0.05$). To explore the effects of an agent’s human likeness and responsiveness on task performance, two $2\times 2$ repeated-measures ANOVAs were performed with the accuracy of the task and the EEG-based attention level as independent variables. To explore the effects of an agent’s human likeness and responsiveness on the experience of human–agent relationships, three $2\times 2$ repeated measures ANOVAs were performed with personal involvement, perceived closeness, and intimacy with agents as independent variables. The results are as follows.

#### Results on the accuracy of task

The results for the accuracy of the task did not reveal any main effects. Neither human-likeness [$F_{(1,15)}=1.756$, $p=0.205$, ${\eta _p}^2=0.105$] nor responsiveness [$F_{(1,15)}=0.850$, $p=0.371$, ${\eta _p}^2=0.054$] significantly affected the accuracy. The interaction effect of human-likeness $\times$ responsiveness was not significant [$F_{(1,15)}=3.768$, $p=0.071$, ${\eta _p}^2=0.201$].

#### Results on the attention

**Figure 6:**
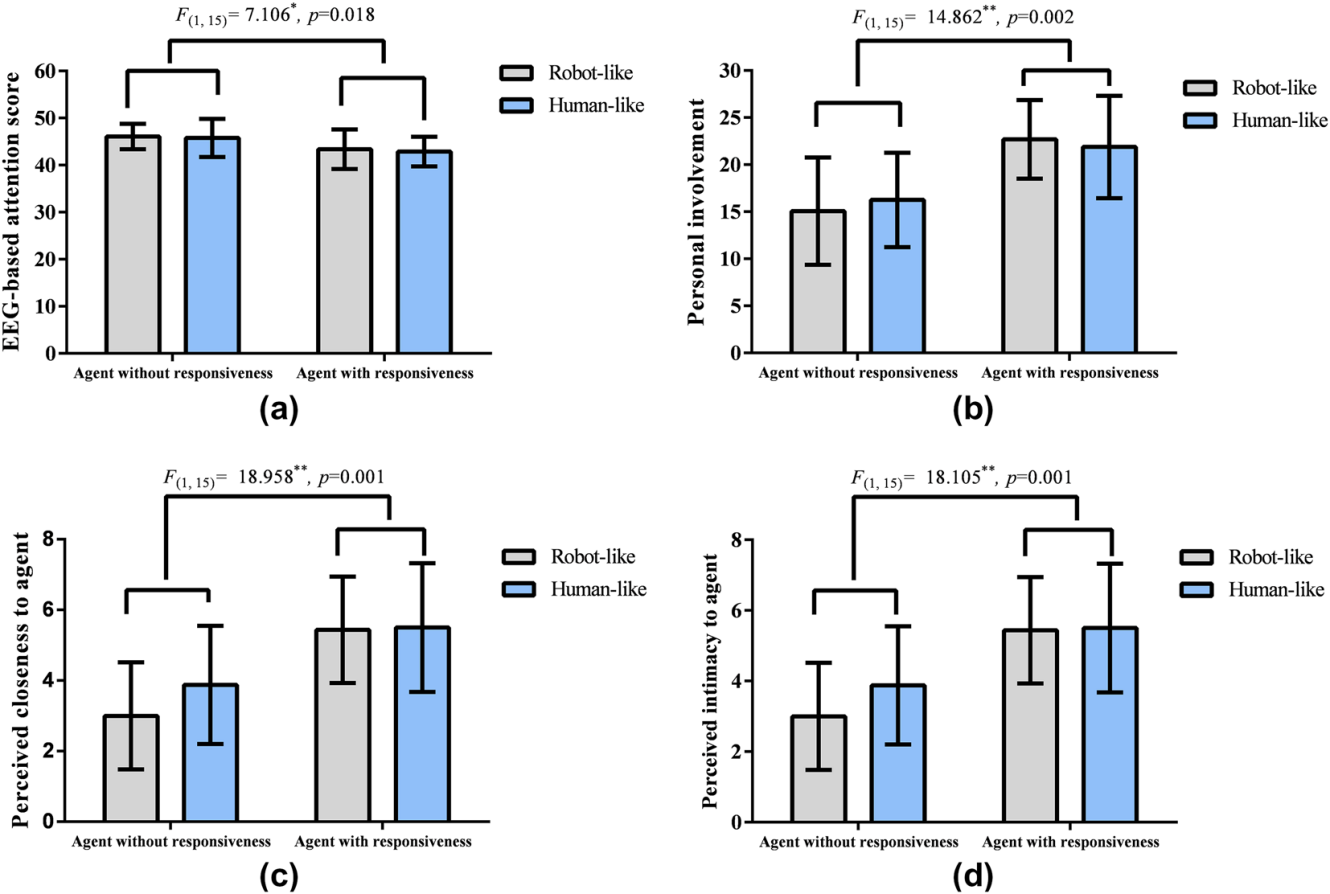
Results on participants’ attention level reported from EEG device (**a**), personal involvement to agent (**b**), perceived closeness (**c**), and intimacy (**d**) in Experiment 1. Participants had a significantly lower level of attention on agents with responsiveness than that without responsiveness ($$*p<0.05$$, $$**p<0.01$$, confidence intervals (CIs) in figures represent 95$$\%$$ CIs. However, participants perceived significantly higher level of involvement, closeness, and intimacy in agents with responsiveness than those in agents without responsiveness.

The results for the EEG-based attention level revealed a significant main effect of responsiveness [$F_{(1,15)}=7.106$, $p=0.018$, ${\eta _p}^2=0.321$] (Fig. 6a in Experiment1). Participants had a higher level of attention to agents without responsiveness ($M=45.94$, $SD=6.35$) than to agents with responsiveness ($M=43.13$, $SD=6.83$).

Neither the main effect of agent’s human-likeness [$F_{(1,15)}=0.066$, $p=0.800$, ${\eta _p}^2=0.004$] nor the interaction effect [$F_{(1,15)}=0.003$, $p=0.096$, ${\eta _p}^2=0.000$] was significant.

#### Results on personal involvement

The results on personal involvement also indicated a significant main effect of responsiveness [$F_{(1,15)}=14.862$, $p=0.002$, ${\eta _p}^2=0.498$] (Fig. 6b in Experiment1). Participants perceived a higher level of involvement with agents with responsiveness ($M=22.28, SD=8.97$) than with agents without responsiveness ($M=15.66, SD=9.93$).

Neither the main effect of the agent’s human-likeness [$F_{(1,15)}=0.043$, $p=0.838$, ${\eta _p}^2=0.003$] nor the interaction effect [$F_{(1,15)}=1.132$, $p=0.304$, ${\eta _p}^2=0.070$] was significant.

#### Results on perceived closeness

The results on perceived closeness revealed a significant main effect of responsiveness [$F_{(1,15)}=18.958$, $p=0.001$, ${\eta _p}^2=0.558$] (Fig. 6c in Experiment1). Specifically, participants perceived a higher level of closeness to agents with responsiveness ($M=5.47$, $SD=3.09$) than agents without responsiveness ($M=3.44$, $SD=2.98$).

Neither the main effect of the agent’s human-likeness [$F_{(1,15)}=0.929$, $p=0.350$, ${\eta _p}^2=0.058$] nor the interaction effect of human-likeness $\times$ responsiveness was significant [$F_{(1,15)}=1.489$, $p=0.241$, ${\eta _p}^2=0.090$].

#### Results on perceived intimacy

Similar to the results on perceived closeness, the results on perceived intimacy revealed a significant main effect of responsiveness [$F_{(1,15)}=18.105$, $p=0.001$, ${\eta _p}^2=0.547$] (Fig. 6d in Experiment1). Specifically, participants perceived a higher level of intimacy with agents with responsiveness ($M=5.66$, $SD=3.06$) than those without responsiveness ($M= 3.38, SD=2.90$).

Neither the main effect of agent’s human-likeness [$F_{(1,15)}=0.396$, $p=0.539$, ${\eta _p}^2=0.026$] nor the interaction effect of social role $\times$ responsiveness [$F_{(1,15)}=0.011$, $p=0.920$, ${\eta _p}^2=0.001$] was significant.

#### Results of the free-response question

The results indicated that 87.5 $\%$ of participants shared their feelings, and all of them used tedious related words (for example, tedious, boring, time-consuming, repetitive, monotonous et al. see Table 1) to describe their feelings regarding the task; the remaining 12.5$\%$ did not share their feelings. These results supported the idea that participants felt tedious about the task.

**Table 1:** Open coding feelings and frequency for each feeling after Experiment 1.

Open coding results (feelings)	Frequency	Open coding results (feelings)	Frequency
Boring	16	Tedious	5
Repetitive	6	Time-consuming	6
Monotonous	5	Sleepy	3
Uninteresting	4	Too easy/simple	2
Focused	3	Calm	2

### Brief discussion of experiment 1

Based on the above results, we conclude that responsiveness is an important cue for agents to improve human–agent relationships. Despite the responsiveness of the agent increased, this did not impact the accuracy of the task, showing that the participants had redundant attention while doing the tedious task. Responsiveness decreased the participants’ attention because they attracted redundant attention to their kind reminders and suggestions. This would not decrease the task performance of participants but helped them develop a positive relationship with agents while working with them. Participants perceived a significantly higher level of involvement with agents with responsiveness, indicating that they felt this type of agent was more important and self-relevant^52^. Accordingly, they felt the agents with responsiveness were more like partners who tried to engage in the task and provided some help, and this characteristic made the participants feel significantly higher closeness and intimacy with those agents.

Based on the effect of responsiveness, we further considered that not only was the responsiveness of agents important in tedious work, but also the strategy to show the social response (i.e., social response strategy) to humans might be important. Moreover, there were no significant main effects of the agent’s human likeness, which should be further examined. To explore this point, we further focused on the social response strategy and presented the next experiment in “Experiment 2: exploring the effect of social response strategy in agent’s responsiveness” section.

## Experiment 2: exploring the effect of social response strategy in agent’s responsiveness

### Purpose

In Experiment 1, the agent’s responsiveness relied on whether the agent provided verbal reminders and suggestions. In this section, we present an experiment that further explores and decomposes this condition into two social response strategies. The agent provides reminders and suggestions when participants make a positive or negative decision. We explore the effect of these two strategies on the human–agent relationship and performance in a tedious task.

### Participants

Another 16 undergraduates, including 12 men and 4 women, who did not participate in Experiment 1 were recruited. Their ages ranged from 19 to 28 years, with an average of 23.31 years ($SD=2.03$ years). All patients had normal hearing and normal or corrected-to-normal vision. Informed consent was obtained from all the participants. This study was conducted in accordance with the Declaration of Helsinki and approved by the Ethics Committee of Human Experimentation at Jining No.1 People’s Hospital.

### Experimental design

Our experiment used a 2 (human-likeness of agent: robot-like, human-like) $\times$ 2 (social response strategies: responding to positive/negative decision) factorial within-subject design. As in Experiment 1, both cues were within-subjects factors. The condition of human likeness was the same as in Experiment 1, while responsiveness was further divided into two conditions according to how the agents responded to the participants. As presented in the “Software of the system” section, participants needed to deal with three events during the internship experience. For each event, participants were presented with different options in the system; these options had different appropriateness for dealing with the events. Making a positive decision meant that participants selected the appropriate option while making a negative decision meant that participants selected the inappropriate option^60^. When experiencing the two social response strategies, the participants were instructed to respond negatively or positively to induce agent responses, and then their perception of the human–agent relationship was measured. In this experiment, we must make the participants conscious of what they have made as a positive/negative choice; thus, it is a better way to make positive and negative responses according to their own understanding.

Specifically, two social response strategies were set as follows:

- Response to positive decision. Participants were told to “respond positively” according to their own understanding. When they made a positive decision, the agent responded by providing comments and suggestions. For example, given the event that a virtual colleague comes to the participant and offers to drop off unfinished work and leave early to have fun together, the participant addresses this by choosing the option of “Decline politely.” The agent then provided comments and suggestions for this decision.
- Response to negative decision. Participants were told to “respond negatively” according to their own understanding. When they made a negative decision, the agent responded by providing comments and suggestions. Following the above example, given the same event, the participant deals with this event by choosing the option of “Accept the colleague’s proposal.” The agent then provided comments and suggestions for this decision.

Given the $2\times 2$ factorial within-subject design, there were four types of agents:

- Virtual robot-like agent with the strategy of responding to positive decisions.
- Virtual robot-like agent with the strategy of responding to negative decisions.
- Virtual human-like agent with the strategy of responding to positive decisions.
- Virtual human-like agent with the strategy of responding to negative decisions.

All the participants were required to experience all four types. To avoid the order effect, the order of experience of the four types was counterbalanced with a Latin square design. There were four sequences, and accordingly, the number of participants was a multiple of four. The dependent variables in the design consisted of two aspects: perceived human–agent relationship (perceived closeness, intimacy, and involvement with the agent) and task performance (the accuracy of the task and the attention level). These were similar to those in Experiment 1.

### Apparatus, environment, measures and procedure

The apparatus, experimental environment, measures of dependent variables, and experimental procedure were similar to those in Experiment 1 (“4Experiment 1: effect of agent’s human-likeness and responsiveness in tedious tasks” section).

### Results

First, K–S tests were performed, and the results supported the assumption of normality ($ps>0.05$). As in Experiment 1, the results of point-biserial correlation did not reveal any significant correlation between gender and each dependent variable ($ps>0.05$), suggesting that gender is not a factor that significantly affects the results. To explore the effects of an agent’s human-likeness and social response strategy on task performance, two $2\times 2$ repeated-measures ANOVAs were performed with the accuracy of the task and the EEG-based attention level as independent variables. Subsequently, to explore the effects of an agent’s human-likeness and social response strategy on the experience of the human–agent relationship, three $2\times 2$ repeated measures ANOVAs were performed with personal involvement, perceived closeness, and intimacy (to agents) as dependent variables. The results are as follows.

#### Results on the accuracy of task

The results for the accuracy of the task did not reveal any significant effects. None of the agents’ human likeness, social response strategy, or interaction effect significantly affected the performance ($ps>0.05$).

#### Results on the attention

The results for the EEG-based attention level did not reveal any main effects. Neither social response strategy [$F_{(1,15)}=4.093$, $p=0.061$, ${\eta _p}^2=0.214$], nor human-likeness [$F_{(1,15)}=4.032$, $p=0.063$, ${\eta _p}^2=0.212$] showed a significant effect. The interaction effect of human-likeness $\times$ social response strategy was not significant [$F_{(1,15)}=0.215$, $p=0.650$, ${\eta _p}^2=0.014$].

#### Results on personal involvement

The results for involvement did not reveal any significant effects. None of the agents’ human likeness, social response strategy, or interaction effects significantly affected personal involvement ($ps>0.05$).

#### Results on perceived closeness

**Figure 7:**
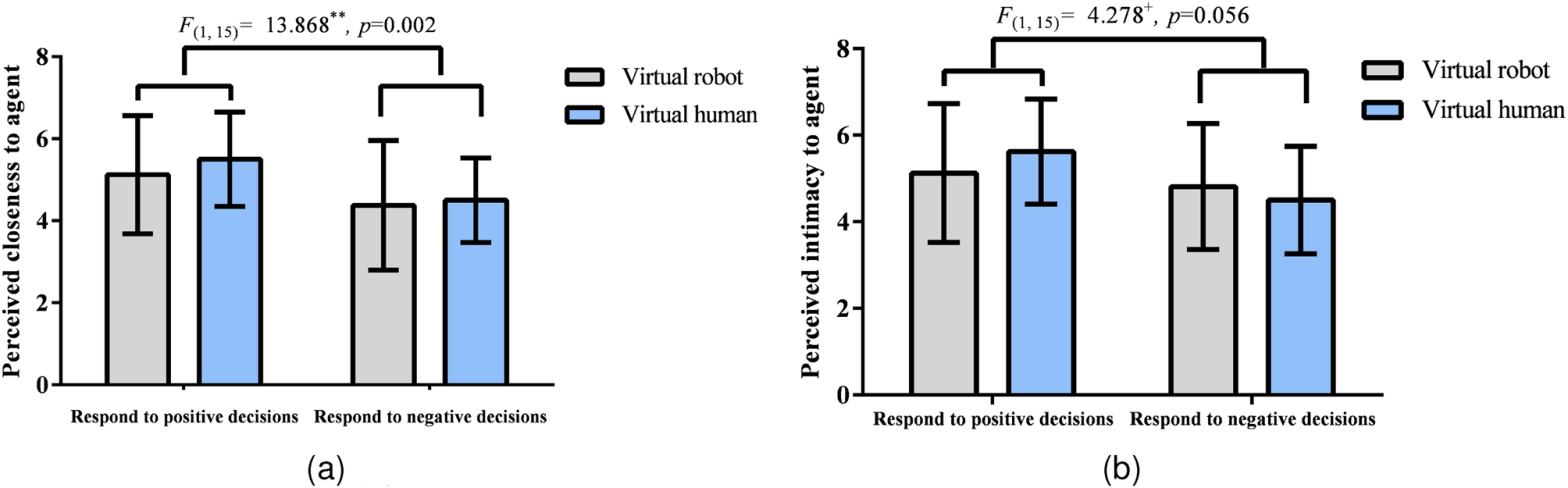
Results on perceived closeness (**a**) and intimacy (**b**) in Experiment 2. Participants perceived a significantly higher level of closeness to agents responding to positive decisions than agents responding to negative decisions. Moreover, participants had a marginally higher level of intimacy with agents responding to positive decisions than agents responding to negative decisions ($$+0.05<p<0.06$$, $$**p<0.001$$, CIs in figures represent 95$$\%$$ CIs).

The social response strategy had a significant main effect on perceived closeness [$F_{(1,15)}=13.868$, $p=0.002$, ${\eta _p}^2=0.480$]. Agents with a strategy of responding to positive decisions led to higher closeness than agents with a strategy of responding to negative decisions (Fig. 7a in experiment2). The main effect of human likeness and interaction effect of human-likeness $\times$ social response strategy was not significant ($ps>0.05$).

#### Results on perceived intimacy

The results for perceived intimacy did not reveal any significant effects. None of the agents’ human likeness, social response strategy, or interaction effects significantly affected perceived intimacy ($ps>0.05$). However, the main effect of the social response strategy could be regarded as marginally significant [$F_{(1,15)}=4.278$, $p=0.056<0.06$, ${\eta _p}^2=0.222$]. Agents with a strategy of responding to positive decisions led to higher intimacy than agents with a strategy of responding to negative decisions (Fig. 7b in experiment2).

#### Results of the free-response question

The results showed that 100% of participants shared their feelings, and all of them used tedious-related words (see Table 2) to describe their feelings regarding the task. These results supported the hypothesis that participants felt tedious about the task.

**Table 2:** Open coding feelings and frequency for each feeling after Experiment 2.

Open coding results (feelings)	Frequency	Open coding results (feelings)	Frequency
Boring	17	Tedious	11
Repetitive	5	Time-consuming	6
Monotonous	3	Sleepy	2
Uninteresting	2	Too easy/simple	2
Cared	2	Calm	3

### Brief discussion of experiment 2

The results of this experiment showed that the social response strategies of the agent were also important cues that significantly affected perceived closeness and marginally affected perceived intimacy without interfering with task performance.

Recent research has shown that highly social agents make users feel more comfortable and less anxious about building interpersonal closeness with them^55^. Agents using a positive social response strategy make participants feel more positive features (for example., more comfortable and less anxious), leading to a significantly higher level of closeness and a marginally higher level of intimacy. Similar to the results in Experiment 1, the agent’s human likeness had no significant effect on the human–agent relationship and task performance. This further supports the result that agents’ vocal feedback ability had a stronger impact on human perception than differences in appearance.

## General discussion

In this study, we investigate the human–agent relationship and task performance in a tedious task when humans interact with virtual agents that have different social cues. We have discussed the results of Experiments 1 and 2 in Sections Brief Discussion of Experiment 1 and Brief Discussion of Experiment 2, respectively. In this section, we present a general discussion of the results of both experiments.

### Attention in tedious task

The results showed that the responsiveness of agents has a significantly negative effect on attention, but has no effect on task accuracy, and has a significantly positive effect on the human–agent relationship.

Tedious tasks are often repetitive and easy to perform. Although certain types of tedious tasks require undistracted attention and are sensitive to relatively brief lapses in attention (such as manual inspection of quality control data^61^), other tedious tasks allow people to allocate their attention^62^. From the perspective of cognitive resources, the participants did not need to spend all their cognitive resources on completing the tedious task^63^. Research on robot agents has shown that they can attract the attention of people by speaking^34^. Therefore, when the agent showed responsiveness by speaking, the participant’s surplus attention was automatically directed to the content of speaking from the ongoing task of sorting parcels. However, reduced attention did not affect the accuracy of tedious tasks.

We believe that the surplus attention allocated to the agents helps participants increase the chance of being involved with them and ultimately leads to positive human–agent relationships, including a better feeling of closeness and intimacy. This feeling of closeness and intimacy with agents can be particularly valuable for people to engage in activities^64^, which are crucial to the development of a long-term relationship (not only short-term effects)^55,65^ and motivation to participate in human–agent collaborative tasks, including tedious activities.

### The effect of responsiveness

The results showed that both the presence of responsiveness and responsive strategies have an important impact on the human–agent relationship.

Regarding the effect of an agent’s responsiveness, previous research has shown that when individuals are faced with certain situations (for example, tedious or stressful situations), only presenting a virtual partner is insufficient; what significantly improves an individual’s emotional experience is the explicit demonstration of attentiveness and responsiveness by the partner^34,55^. Providing appropriate verbal/nonverbal feedback from agents helps users feel cared for and understood, leading to a high degree of acceptance of the agent^4,66^. Therefore, agents with responsiveness can facilitate a sense of rapport and personal involvement through which users can more easily feel closeness and intimacy in human–agent interaction^67–69^.

Regarding the effect of different social response strategies on human–agent relationships, agents using the strategy of responding to positive decisions may be perceived with a more positive impression of social perception. Interpersonal feedback with a positive tendency may increase participants’ motivation and feelings of competence^12^, making them feel more comfortable and less anxious about building interpersonal closeness with the agents^55^. Consequently, this strategy leads to higher interpersonal closeness and intimacy. By comparison, although agents using the strategy of responding to negative decisions also provide rational suggestions, this might still lead to negative feelings and perceptions of participants, leading to lower closeness and intimacy.

### The effect of agent’s human-likeness

It is worth noting that human likeness is not a social cue that significantly affects human–agent relationships and task performance in this study.

Previous studies on social robots have shown that more human-like features (e.g., body shape, face, and voice) make people perceive them as more human-like. This perceived human likeness is an important determinant of human responses to social robots and has been shown to have a positive effect on human–robot interactions^35,36^. Our results are inconsistent with these findings; one possible reason is that the agents in this study are all cartoon style which may weaken the potential effect of human likeness. Another possible reason is that there are no sensitive questionnaires on the agent’s appearance included in this paper. Therefore, the effect of human likeness on the human–agent relationship should be further examined in future work.

Previous research on human–robot interaction shows that dialogue ability has a greater impact on humans’ mental model than physical appearance^70^, which matches our findings that responsiveness, rather than human-like physical qualities, is key for cultivating human–agent relationships.

### Limitations and future work

This study had some limitations. First, there was no questionnaire on the agent’s appearance, which is a limitation in exploring the potential effect of an agent’s human likeness. Including the Godspeed questionnaire^71^ as a potentially more sensitive tool, our next step of the research will further examine the potential effect of the agent’s human likeness using well-known human–agent interaction questionnaires. Second, many more males than females were recruited in both experiments. Although gender is not a factor that significantly affects the results of this study, it would be better to effectively control this factor. We will enroll a larger sample and well control the individual factors in future work. Third, this study used the CAVE system^38^ to create an immersive VR experimental environment. Although wearing a VR head-mounted display (HMD)^72^ can also create an immersive environment and is more accessible to researchers, two considerations halt this study from choosing it: (1) participants need to wear an EEG headband, which may interfere with VR HMD while wearing; (2) the experimental time in this study is slightly long (12 min/round) for VR HMD, which may lead to visual fatigue or VR sickness, but this is not a serious problem for the CAVE system^72^. We believe that if these two considerations can be further optimized, our designed task can be expanded to VR HMD, which will be more available and beneficial to other researchers in future studies.

## Conclusions

In this study, we explore the effect of agents’ social cues on the human–agent relationship and performance in the tedious task of parcel sorting. Two social cues, that is, human likeness and responsiveness of the agent, were examined by constructing a virtual parcel-sorting task and a prototype system. Our results show that:

- The responsiveness of the agent has a significant effect on the user experience in a tedious task. Responsiveness and an appropriate social response strategy to show responsiveness can greatly improve the human–agent relationship without compromising the performance efficiency of tedious tasks.
- Compared with human likeness, the responsiveness of the agent is more important for building a human–agent relationship.

In summary, agents that have a higher level of responsiveness and use appropriate response strategies to show responsiveness to users may be most conducive to building good human–agent relationships in tedious tasks.

## Supplementary Information

Supplementary Information 1.

Supplementary Information 2.

Supplementary Video 1.

Supplementary Table 1.

## Data Availability

All data generated or analyzed during this study are included in this published article [and its supplementary information files].
