# Supplementary material for: Exploring user experience and performance of a tedious task through human–agent relationship
Source: Sci Rep. 2023 Feb 21;13:2995. doi: 10.1038/s41598-023-29874-5 (PMC9944929; doi:10.1038/s41598-023-29874-5)
Supplement: Supplementary file 4 — Supplementary Table 1. [file 41598_2023_29874_MOESM4_ESM.pdf]

The three events, what participants could respond with and the corresponding feedbacks from the agent are detailed shown in the following table.

Table 1. Details of the three events (including the events description, the user's choices, and the corresponding feedback from the agent)

Event topics	Description of the events	Alternative choices	Feedbacks from the agent
Event 1: Leave early from work.	A virtual colleague comes to the participant and offers to drop off unfinished work and leave early to have fun together, and the participant needs to deal with this event by choosing an option.	Choice 1: Give a stern rebuff	Your action may make your colleagues be mad with you. Your work principle is correct, but it may be better if you try to use a gentler way first.
		Choice 2: Decline politely.	I think you handled it properly. This not only complies with the company's regulations, but also won't break your relationships with the colleague.
		Choice 3: Accept the colleague's proposal.	Friendly reminder to you that your decision is against the company's regulations. One of the basic requirements of our company for employees is to comply with the regulations.
Event 2: Colleague is criticized	Your colleague made a serious mistake in his work, and the superior severely criticized him in front of you.	Choice 1: Don't take any action	In this way, the colleague may feel frustrated. He may need encouragement and advice now, and you can try showing some care to him. This may also be helpful for you to develop positive interpersonal relationships.
		Choice 2: Actively care and comfort the colleague	I support your decision and action. Actively expressing care for our colleagues may help to enhance your interpersonal relationships.
		Choice 3: Tell others on this matter.	Your action may make your colleagues mad at you. It is unfriendly to spread others' embarrassing stories, which is easy to arouse his dislike of you.
Event 3: Talk to your superior	Your superior wants to investigate the typical problems in the workplace, and you have the opportunity to have a direct talk with him.	Choice 1: Highlight problems truthfully and provide constructive suggestions.	I support your action. You should seek truth from facts and focus more on the real problem so that your superiors can see your potential in finding and solving problems.
		Choice 2: Report something uncertain or rumor.	Your superior may think you are unreliable. We should seek truth from facts when reporting the situation to the superior. Spreading rumors to the superior is not beneficial to your work.
		Choice 3: Use the opportunity to talk about and praise yourself.	This may not be what the superiors want to hear. It is not very wise to be eager to praise yourself in front of more capable people. You'd better focus on your work first and maintain a modest attitude.
